# Ischemia with Non-Obstructive Coronary Artery Disease: Sex-Based Differences in Pathophysiology, Clinical Presentation, and Prognosis

**DOI:** 10.3390/jcm14165764

**Published:** 2025-08-14

**Authors:** Filippo Luca Gurgoglione, Giorgio Benatti, Andrea Denegri, Emilia Solinas, Iacopo Tadonio, Mattia De Gregorio, Laura Torlai Triglia, Davide Donelli, Marco Covani, Gabriella Dallaglio, Federico Barocelli, Giulia Magnani, Michele Russo, Luigi Vignali, Giampaolo Niccoli

**Affiliations:** 1 Division of Cardiology, Parma University Hospital, 43126 Parma, Italy; filippolucagurgoglione@gmail.com (F.L.G.); giorgiobenatti88@gmail.com (G.B.); adenegri@ao.pr.it (A.D.); itadonio@ao.pr.it (I.T.); mattia.de.gregorio@gmail.com (M.D.G.); laura.torlait@gmail.com (L.T.T.); donelli.davide@gmail.com (D.D.); marco.covani@unipr.it (M.C.); gabriella.dallaglio@unipr.it (G.D.); fbarocelli@ao.pr.it (F.B.); giuliamagnani.cardio@gmail.com (G.M.); luvignali@ao.pr.it (L.V.); 2 Department of Cardiology, SS. Annunziata Hospital, ASL2 Abruzzo, 66013 Chieti, Italy; michelerusso0509@gmail.com

**Keywords:** coronary microvascular dysfunction, gender differences, prognosis

## Abstract

Ischemia with non-obstructive coronary arteries (INOCA) is a chronic coronary condition associated with poor prognosis and reduced quality of life. The increasing use of invasive assessments of microcirculatory function and provocative spasm testing has significantly advanced the understanding of INOCA’s pathophysiology, which ranges from coronary microvascular dysfunction to vasomotor disorders. However, the optimal management and therapeutic approach for INOCA remain to be determined. Significant sex-based differences in the pathophysiology of INOCA have been reported, resulting in variations in prevalence, phenotype, and natural history between male and female patients. The aim of this narrative review is to provide a comprehensive overview of the sex-specific pathophysiological mechanisms underlying INOCA and to summarize the differences in INOCA phenotype and prognosis. Additionally, we will explore the current knowledge on management and therapy, with the goal of advancing towards sex-based personalized treatment strategies for INOCA.

## 1. Introduction

Ischemic heart disease is the leading cause of morbidity and mortality worldwide. A large proportion of patients undergoing invasive coronary angiography for anginal symptoms do not exhibit obstructive coronary artery disease (CAD). However, coronary angiography has a limited spatial resolution (approximately 0.3 mm) and is unable to visualize distal branches and arterioles, which play a central role in regulating myocardial blood flow [1].

Up to 70% of patients with ischemia with non-obstructive coronary artery disease (INOCA) present functional disorders, encompassing coronary microvascular dysfunction (CMD) and coronary artery spasm (CAS) [2]. Far from being a benign condition, INOCA is associated with an elevated risk of adverse cardiovascular events, impaired quality of life, recurrent hospitalizations, repeated coronary angiographies, and increased healthcare costs [3]. As these conditions can only be ascertained with additional specific functional and provocative tests, they are often misdiagnosed as non-cardiac disorders and undertreated.

In recent years, the implementation of a standardized invasive diagnostic protocol has been endorsed by the latest European guidelines [4], demonstrating unique potential to evaluate both epicardial and microvascular compartments. This protocol employs a dedicated pressure wire with thermodilution techniques to assess CMD, and intracoronary acetylcholine (ACh) administration to evaluate the CAS [5,6]. The use of this protocol facilitates tailored treatment strategies [7] and has been shown to improve both quality of life and prognosis [8,9].

Robust evidence indicates that women have a higher prevalence of non-obstructive CAD and coronary functional disorders compared with men [10]. Clear sex-based differences have also been documented in the setting of obstructive CAD [11]. Therefore, a sex-specific understanding of this disease is crucial for identifying differences in prevalence, pathophysiology, clinical presentation, and treatment response, ultimately enabling more tailored, patient-centered care.

The following search strategy was used to identify pertinent studies: ((coronary microvascular dysfunction[Title/Abstract] OR coronary spasm[Title/Abstract] OR microvascular angina[Title/Abstract]) AND (sex[Title/Abstract] OR gender[Title/Abstract] OR female[Title/Abstract] OR wom*[Title/Abstract])).

## 2. INOCA Overview

### 2.1. Prevalence

INOCA is a clinical condition characterized by signs and symptoms of myocardial ischemia in the absence of significant obstructive CAD on coronary angiography [2]. Recent studies estimate that up to 50% of patients who undergo coronary angiography for angina symptoms do not exhibit obstructive CAD [1]. The prevalence of INOCA also increases with age and is more commonly observed in individuals with comorbidities such as hypertension, diabetes, dyslipidemia, obesity, and systemic inflammation [12].

### 2.2. Pathophysiology

The pathophysiology of INOCA is complex and multifactorial, often involving abnormalities in coronary blood flow regulation and vasomotor function rather than fixed obstructive lesions [13]. The primary mechanisms underlying INOCA include the following: (1) CMD; (2) endothelial dysfunction; (3) CAS; (4) autonomic imbalance and inflammation.

CMD refers to the impaired regulation of blood flow in the small resistance vessels (<200 μm) of the coronary circulation, where structural or functional abnormalities prevent adequate vasodilation during increased myocardial demand, leading to ischemia [14]. Endothelial dysfunction is the result of impaired vasodilation, increased oxidative stress, and pro-inflammatory states [15].

The CAS involves the transient vasoconstriction of epicardial and/or microvascular coronary arteries, primarily related to endothelial dysfunction and increased reactivity of vascular smooth muscle [16]. Additionally, chronic inflammation can exacerbate microvascular and vasomotor dysfunction as well as increase platelet reactivity. Emerging evidence also links INOCA to microvascular inflammation and heightened platelet activity and air pollution [17,18]. Notably, these mechanisms are not mutually exclusive and often coexist, complicating clinical presentation and management.

### 2.3. Phenotypes

CMD is traditionally classified into two phenotypes: (1) structural CMD, characterized by the remodeling of small resistance arterioles with hypertrophic inward changes, increased stiffness accompanied by perivascular fibrosis, and capillary rarefaction; and (2) functional CMD, defined by the dysregulation of small arteriole tone, with a predominance of vasoconstrictive over vasodilatory agents. Invasive functional testing enables the diagnosis and phenotypic categorization of CMD. CMD is defined by a reduced coronary flow reserve (CFR < 2) and is further classified as structural when the index of microvascular resistance (IMR) is abnormal (>25), or as functional when the IMR is ≤25 [5,6].

The CAS is typically characterized by rest angina, with or without transient ST-segment elevation and often requires multimodality diagnostic assessment [19,20,21]. Notably, the CAS can be provoked by invasive spasm testing with ACh [22]. Intracoronary administration of ACh elicits the CAS in predisposed subjects with more than 90% sensitivity and 99% specificity [23,24]. The Coronary Vasomotion Disorders International Study (COVADIS) group have proposed specific indications for invasive provocative testing and established the diagnostic criteria for the CAS. Specifically, epicardial CAS is characterized by a ≥90% reduction in epicardial coronary diameter, accompanied by reproduction of the patient’s symptoms and ischemic ECG changes, while microvascular spasm is defined by typical angina and ischemic ECG changes in the absence of significant epicardial coronary constriction [25,26]. It is not uncommon for these phenotypes to coexist (mixed phenotype), complicating management and necessitating combined therapeutic strategies. Some individuals present with ischemic symptoms in whom extensive diagnostic workups, including coronary function testing, fail to identify a specific mechanism. Finally, certain patient populations—such as those with systemic autoimmune diseases—often exhibit microvascular dysfunction as the predominant underlying mechanism, primarily driven by systemic endothelial inflammation [27].

## 3. Pathophysiology of INOCA in Women

Women exhibit distinct anatomic and functional characteristics of the coronary microcirculation that may contribute to the higher prevalence of CMD. Notably, women have smaller and thinner epicardial coronary arteries than men, even after adjusting for left ventricular mass and body composition, which may predispose to adverse arterial remodeling and impaired vasodilatory capacity [28]. Additionally, women demonstrate microcirculatory functional abnormalities, as evidenced by higher resting coronary blood flow compared to men, likely attributable to the dysregulation of the nitric oxide metabolism and autonomic nervous system activity [29].

Furthermore, women are more likely to present non-traditional cardiovascular risk factors (NTCVRF), which are associated with accelerated premature atherosclerosis [30], spontaneous coronary artery dissection [31], but also with INOCA. Both sex-predominant NTCVRF and gender-related NTCVRF are described [32]. The first group comprises several risk factors that collectively contribute to the higher prevalence of CMD in women. Hormonal factors, particularly estrogen fluctuations, play a pivotal role in the increased incidence of CMD during the peri- and postmenopausal stages [33]. Estrogens are critical regulators of vascular tone, modulating both endothelial and vascular smooth muscle cell (VSMC) function [34]. At the endothelial level, estrogens enhance the release of vasodilatory mediators, such as nitric oxide, while suppressing the production of vasoconstrictive agents and pro-inflammatory cytokines. At the VSMC level, estrogens inhibit transdifferentiation into fibroblasts, thereby mitigating intimal thickening, and the progression of endothelial dysfunction [35]. Consequently, the postmenopausal decline in estrogen levels represents a key mechanistic driver in the pathogenesis of CMD.

Furthermore, women are more frequently affected by chronic systemic inflammatory and autoimmune diseases. These conditions predispose to both systemic and coronary microvascular dysfunction by promoting endothelial inflammation and a sustained vasoconstrictive state [36]. Pregnancy-related disorders, such as gestational diabetes, pre-eclampsia, and eclampsia, also contribute to CMD by inducing hypertensive and hyperglycemia-mediated endothelial injury, further exacerbated by hormonal fluctuations [37].

Gender-related NTCVRF include challenging stress-coping strategies, a higher prevalence of depression, anxiety syndrome, elevated psychological stress, post-traumatic stress disorders, and also type D personality. Additionally, psychosocial disadvantages, like being the sole caregiver, widowhood, low economic status, poor healthcare literacy or intimate partner violence, affect women more often than men. These factors are strongly associated with poorer quality of life and higher levels of angina symptomatology in women compared with male controls [38]. The underlying pathophysiology is linked to chronic distress of the heart-brain system, a paradigm of the psycho-neuro-endocrine-immunological model of system activation. Specifically, heightened basal sympathetic tone and hyperactivation of the hypothalamic–pituitary axis elevate cortisol and catecholamine levels, leading to vasoconstriction, increased heart rate, enhanced platelet activation, and elevated circulating pro-inflammatory cytokines [39]. Beyond the chronic stress effects, a pivotal role is played by the imbalance between the two branches of the autonomic nervous system, particularly in aging women. During perimenopause, sympathetic tone predominates, impairing coronary flow and promoting inflammation, which ultimately culminates in endothelial dysfunction. Moreover, low heart rate variability has been shown to independently predict myocardial ischemia, underscoring the significance of sympatho-vagal imbalance in INOCA [40]. In the CANS study (Cardiac Autonomic Nervous System), women with INOCA exhibited greater microvascular and peripheral vasoconstriction in response to arithmetic mental stress compared to age-matched controls without anginal symptoms. Both groups demonstrated similar increases in blood pressure and heart rate during mental stress; however, the INOCA group experienced higher thoracic pain and microvascular reactivity. Notably, more than a quarter of women with INOCA also showed abnormal autonomic system activity, as detected by meta-iodobenzylguanidine cardiac imaging, with adrenergic nervous system dysfunction predicting worse clinical outcomes [41].

## 4. CMD in Women vs. Men: Prevalence and Prognosis

The majority of evidence reported a higher prevalence of CMD, with lower CFR and high IMR values, in women compared to men [42,43,44,45,46,47,48,49,50].

A large, comprehensive meta-analysis pooled together data from 37 studies reporting rates of CMD in patients with non-obstructive CAD, using both invasive (n = 23) and non-invasive (n = 14) diagnostic tools. The overall prevalence of CMD was 41%, with no significant differences concerning the diagnostic method (i.e., invasive vs. non-invasive) or the CFR threshold used (i.e., ≤2.5 or ≤2.0). The risk of testing positive for CMD was 1.45 times higher for women [51].

More recently, a subanalysis on sex differences from the ILIAS registry confirmed a significantly higher prevalence of CMD among women with non-obstructive CAD compared to men (46.4% vs. 43%, respectively, *p* < 0.01) [52].

Once considered a benign condition, INOCA has been demonstrated by multiple studies to be associated with poor clinical outcomes compared to a reference population without objectification of ischemia or anginal symptoms [53,54,55,56].

Patients with anginal symptoms with non-obstructive CAD are more likely to suffer from impaired physical, mental and social health, quality of life, and have a higher risk of undergoing recurrent hospitalizations and repeated coronary angiograms, resulting in increased healthcare costs [53]. These issues disproportionately affect women. The WISE study showed that women often report poor functional capacity and persistent chest pain, significantly limiting daily activities [57]. Psychological conditions such as anxiety, panic disorder, and depression are also more common in women [58]. Many are forced into early retirement, and nearly one-third must change jobs due to physical limitations [59].

A lower CFR and higher IMR, along with frequent abnormalities in vascular tone regulation, may contribute to the poorer quality of life seen in women compared to men [29,44]. Autonomic imbalance and nociceptive fiber dysregulation may further amplify sex-related differences in symptom perception [39].

Moreover, the greater prevalence of psychological disorders in women may directly contribute to CMD pathophysiology by promoting sympathetic overactivity, low-grade inflammation, and endothelial dysfunction [59].

In addition, hard cardiovascular outcomes are affected as well [53].

In the WISE study, CFR was an independent predictor of adverse cardiovascular events (death, non-fatal myocardial infarction, non-fatal stroke, or hospital stay for heart failure) among the overall women population, regardless of the presence of obstructive CAD [54].

Numerous subsequent studies have explored this possible association with variable results.

Recently, Odanovic et al. conducted an up-to-date meta-analysis pooling data from 39 studies reporting outcomes for CMD. At a median follow-up of 6.6 years, patients with a reduced CFR exhibited the worst prognosis within the entire INOCA spectrum, with 5.7 times higher risk of death and myocardial infarction (incidence 4.7 (95% CI 2.0–8.4)/100 patient-years, *p* < 0.01) compared to patients with only non-invasive stress tests [55].

Indeed, the underlying mechanisms of adverse outcomes are poorly understood. To date, no established medical therapies have demonstrated effectiveness in improving the prognosis of these patients. Whether reduced CFR represents a causal factor or merely a marker of adverse outcomes is yet to be conclusively determined and requires further research (Table 1).

## 5. Vasomotor Disorders in Women vs. Men: Prevalence and Prognosis

Clear-cut sex-based differences have been observed in clinical characteristics, CAS features and prognosis among patients with suspected or confirmed CAS [60,61,62,63,64,65,66,67,68,69,70,71].

However, racial differences between European and Asiatic studies, as well as variations in invasive provocative test protocols and diagnostic criteria for CAS, contribute to significant heterogeneity across studies. For instance, the threshold for epicardial coronary diameter reduction in response to ACh differs among studies, ranging from >70% [63], >75% [64], to >95% [61], compared to the relaxed state after intracoronary nitroglycerin infusion. Additionally, a Japanese study assessed lactate production and reduction in coronary blood flow for the diagnosis of microvascular CAS rather than the COVADIS criteria [66]. Notably, most studies examining sex-based differences in CAS have reported data exclusively on epicardial CAS, with only few studies focusing on microvascular CAS [66,67,71].

Compelling evidence indicates a higher prevalence of epicardial CAS in men, with odds ratios ranging from 1.24 to 1.70, compared to women. Moreover, sex-based topographic differences have been described as follows: women are more likely to present with distal and diffuse CAS, whereas men predominantly exhibit focal, proximal, and multivessel CAS [64,68,70]. Angiographic differences between men and women may help explain these findings. Men are more likely to have non-obstructive CAD, often with more vulnerable plaque features [72,73], and a history of prior percutaneous coronary intervention with drug-eluting stents [74]. Previous studies have shown a predisposition for CAS in coronary segments harboring non-obstructive plaques, likely attributable to underlying endothelial dysfunction. Moreover, stent implantation may promote the CAS through the release of antiproliferative drugs and hypersensitivity reactions to the metallic struts and polymer coatings [74]. An additional contributing factor is the higher prevalence of myocardial bridging (MB) in men, as reported by Park et al. [70]. A strong association between MB and the CAS has been documented, likely reflecting enhanced local vascular reactivity to vasoconstrictor stimuli within MB segments, mediated by endothelial dysfunction [75].

Furthermore, men appear to be more sensitive to ACh, as the mean maximum ACh dose required to provoke a positive spasm was notably higher in females than in males in both coronary arteries, particularly in Japanese populations [76]. In contrast to these findings, Aziz et al. reported a significantly higher frequency of epicardial spasm in females compared to males (27.8% vs. 22.9%, *p* < 0.05). However, their study population was younger and excluded patients with a history of CAD, which is typically more prevalent in males. Notably, their definition of a positive test for epicardial CAS (>70% vasoconstriction) does not align with the COVADIS definition [63].

On the other hand, women consistently exhibit a higher prevalence of microvascular CAS compared to men, with reported odds ratios ranging from 1.38 to 7.16 [61,62,66]. Anatomical peculiarities in women, such as smaller vessel diameters, thinner arterial walls, and hormonal influences, may underpin these observations [28,29].

These sex-based differences also have important prognostic implications. Several Japanese studies have consistently reported comparable outcomes between sexes among patients with epicardial CAS admitted with INOCA [61,62,64,69,70].

Park et al. observed favorable outcomes at the 5-year follow-up after positive ACh test, with MACE incidences of 0.6% in women and 1.2% in men (*p* = 0.201). The presence of non-obstructive CAD was a predictor of MACE and recurrent angina in both sexes [70]. Similarly, Kawana et al. reported comparable prognoses between sexes, with MACE rates of 6.1% in men and 5.6% in women over a median follow-up of 32 months. Interestingly, predictors of MACE differed by sex: in men, smoking habit, a history of myocardial infarction, and non-obstructive CAD were significant predictors, whereas in women, age and the onset of electrical abnormalities were significant predictors. Notably, younger women (<50 years) exhibited significantly lower MACE-free survival compared to older women [62]. The higher prevalence of smoking among younger women may enhance Rho-kinase activity, promoting CAS and potentially leading to life-threatening arrhythmias [77].

The study by Kim et al. also reported an association between lower body mass index (BMI) and worse outcomes [65], likely related to reduced estrogen production in women with low BMI [78].

A recent study by Rinaldi et al. extended these findings by including also patients with MINOCA. At a median follow-up of 22 months, the authors observed a similar rate of MACCE between women and men, both in suspected CAS (9.5% vs. 11.1%, *p* = 0.49) and in positive ACh tests (9.6% vs. 17.2%, *p* = 0.08). Likewise, angina recurrence rates were comparable between women and men in the suspected CAS population (33.8% vs. 24.6%, *p* = 0.06) and in the positive ACh test (41.8% vs. 32.8%, *p* = 0.24). Interestingly, when evaluating each sex separately, they found that a positive ACh test was associated with a higher rate of angina recurrence in women and a higher rate of MACCE in men [71]. This difference may be attributed to the higher prevalence of epicardial CAS in men [70], and the higher prevalence of microvascular CAS in women [51]. Of relevance, the higher risk of MACE observed in men compared to women with epicardial CAS may be attributed to the more frequent coexistence of significant epicardial atherosclerosis and a greater burden of cardiovascular risk factors. These factors may contribute to the development of accelerated atherosclerosis, thereby increasing the risk of myocardial infarction and cardiac death [62,64,71] (Table 2).

## 6. Potential Therapeutic Implications and Future Perspectives

**Figure 1:**
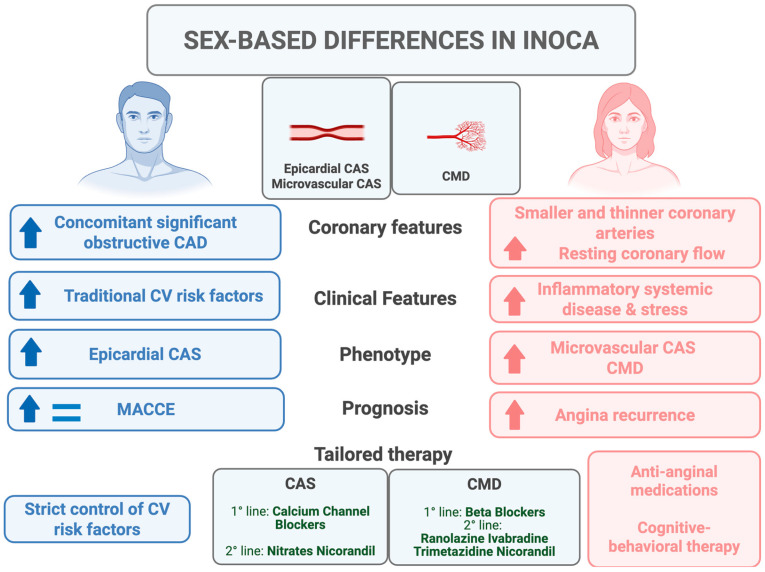
Main differences between women versus men in CMD. Abbreviations. CAD: coronary artery disease; CAS: coronary artery spasm; CMD: coronary microvascular dysfunction; CV: cardiovascular; INOCA: ischemia with non-obstructive coronary artery disease; MACCE: major adverse cardiovascular and cerebrovascular events. Figure was created with BioRender.com.

This overview of the current literature suggests different clinical, phenotype, and prognostic implications between sexes among patients with INOCA (Figure 1).

Men present with a higher burden of traditional cardiovascular risk factors, a higher prevalence of epicardial CAS and a higher risk of MACCE at follow-up [61,67,68,69,70,71]. Conversely, women present with a higher prevalence of sex-specific risk factors (e.g., polycystic ovary syndrome, autoimmune diseases, gestational diabetes, gestational hypertension), CMD and microvascular CAS, and are at higher risk of poor quality of life and recurrent angina at follow-up [63,66,67,71].

A standardized invasive diagnostic protocol is essential for accurate diagnosis. Yet, despite guideline recommendations [2], it remains underused in clinical practice and often reserved for therapy-refractory cases. Historically, limited data on clinical utility and concerns about procedural risks have restricted its use. However, recent studies have confirmed its diagnostic value, reporting only rare adverse events [2,13,16,22,26]. When clinically indicated, the omission of testing is no longer justified. The routine use of non-invasive diagnostic modalities warrants further investigation. Among these, cardiac positron emission tomography (PET) is currently the most accurate non-invasive imaging technique for diagnosing CMD, due to its ability to precisely quantify CFR. A CFR < 2, as measured by PET, is associated with adverse clinical outcomes [79]. Cardiac magnetic resonance imaging serves as a robust alternative, demonstrating excellent diagnostic accuracy for CMD and showing strong concordance with invasive reference standards [80]. Furthermore, novel diagnostic modalities are under evaluation for CMD. For example, machine learning algorithms based on non-invasive electrocardiograms have demonstrated good accuracy in detecting CMD [81,82].

Importantly, the use of a standardized protocol may help guide tailored diagnostic and therapeutic strategies. According to current clinical guidelines [3,9], beta-blockers are recommended as the first-line pharmacologic therapy for CMD. For patients who remain symptomatic despite initial treatment, second-line agents—including nicorandil, ranolazine, and ivabradine—may be considered. In cases of CAS, calcium channel blockers remain the mainstay of therapy, while nitrates serve as a second-line option when vasospastic symptoms persist. Additionally, statin therapy is recommended for all patients with INOCA.

Several emerging therapies show potential in improving outcomes and quality of life in patients with CMD [83]. Among pharmacologic approaches, endothelin receptor antagonists have demonstrated the potential to improve microvascular endothelial function [84]. However, the current evidence of clinical benefit is limited, and further randomized studies are needed. CD34^+^ stem cell therapy, which promotes angiogenesis and endothelial repair, has also shown promise. Two recent trials reported reduced angina frequency following the intracoronary infusion of CD34^+^ cells [85,86]. The ongoing FREEDOM trial (ClinicalTrials.gov Identifier: NCT04614467) will further evaluate its efficacy and safety in patients with CMD and refractory angina. The coronary sinus reducer, a percutaneously implanted stainless-steel mesh device designed to enhance myocardial perfusion, has demonstrated improvements in quality of life and reductions in IMR in early studies [87,88]. Its clinical utility is currently being assessed in the phase III COSIRA-2 trial (ClinicalTrials.gov Identifier: NCT05102019). Lastly, cognitive-behavioral therapy has been proven to promote improvements in psychological well-being, physical functioning, anginal burden, and exercise tolerance [89].

These findings pave the way toward a sex-specific management of patients with INOCA. Indeed, male patients might require high-dose calcium channel blockers and the aggressive management of traditional cardiovascular risk factors. For female patients, the use of anti-anginal medications, as well as long-term lifestyle modifications and the implementation of new broader therapeutic strategies, including psychological support, should be encouraged [2,4].

Future randomized studies are warranted to address the prognostic benefit of the sex-specific management of INOCA patients.

## Figures and Tables

**Table 1:** Main studies reporting sex-based differences in CMD prevalence, invasive features, and prognosis.

First Author, Year, Date, Reference	Study Population	Proportion of Females	Endpoints	Results [Women vs. Men]
Kobayashi et al., 2015 [44]	157 ANOCA patients	74.5%	CMD: CFR < 2.0 and/or IMR ≥ 25	Abnormal CFR: 8.6% vs. 0% * CFR: 3.8 ± 1.6 vs. 4.8 ± 1.9 * Abnormal IMR: 28.2% vs. 15%
Sara et al., 2016 [45]	926 ANOCA/INOCA patients	74.7%	CMD: CFR ≤ 2.5	CMD: 30.6% vs. 29.9%
Ford et al., 2019 [46]	187 INOCA patients	67.9%	MVA: IMR ≥ 25 and/or CFR < 2.0 and/or microvascular spasm	MVA: 74.8% vs. 65%
Pargaonkar et al., 2019 [47]	155 INOCA patients	76.8%	CMD: IMR ≥ 25	CMD: 21% vs. 19.4%
Rahman et al., 2019 [48]	85 ANOCA patients	77.7%	CMD: CFR < 2.5	CMD: 59% vs. 31.6%
Kumar et al., 2020 [49]	163 INOCA and UA patients	48.5%	CMD: CFR < 2.5 and/or HMR ≥ 2 and/or ≤50% change in coronary blood flow with Ach	CFR: 2.2 ± 0.8 vs. 2.5 ± 1.1 *
Chung et al., 2020 [50]	434 ANOCA patients	30.7%	CMD: CFR ≤ 2.0 and/or IMR ≥ 23	CMD: 46.6% vs. 39.5% CFR: 2.69 vs. 3.20 * IMR: 17.9 vs. 17.1 5-year MACE rate:1.1% vs. 5.5% *

Table legend. Ach: acetylcholine; ANOCA: angina with non-obstructive coronary arteries; CFR: coronary flow reserve; CMD: coronary microvascular dysfunction; HMR: hyperemic microvascular resistance; IMR: index of microvascular resistance; INOCA: ischemia with non-obstructive coronary arteries; MACE: major adverse cardiovascular events; MVA: microvascular angina UA: unstable angina. * *p*-value < 0.05.

**Table 2:** Coronary vasomotor disorders: prevalence and prognosis in women versus men.

First Author, Date, Reference	Study Population	Proportion of Females	Endpoints	Results
Lee et al., 2009 [61]	104 ANOCA patients	20.2%	Epicardial CAS: >95% diameter reduction + chest pain + ischemic ECG changes	Men have more CAS in the RCA, women in the LAD.
Ohba et al., 2012 [66]	370 ANOCA patients	57.0%	Epicardial CAS: >90% diameter reduction + chest pain + ischemic ECG changes. Microvascular CAS: transcardiac lactate production + decreased quantitative coronary blood flow	Female sex was independently correlated with the presence of microvascular CAS. Patients with microvascular CAS exhibited a good prognosis after treatment with CCBs.
Kawana et al., 2013 [62]	1429 VSA patients	23.7%	Epicardial CAS: >90% spasm + chest pain + ischemic ECG changes	No significant sex-based differences in 5-year MACE-free survival. The long-term prognosis was lowest in the young female group.
Aziz et al., 2017 [63]	1379 ANOCA patients	42%	Epicardial CAS: >75% diameter reduction + chest pain + ischemic ECG changes. Microvascular CAS: <75% diameter reduction + chest pain + ischemic ECG changes	Women had a higher prevalence of both epicardial and microvascular CAS.
Sueda et al., 2021 [68]	917 VSA patients	80.4%	Epicardial coronary spasm: >90% spasm + typical chest pain + ECG changes	Women had a higher prevalence of diffuse and combined CAS. Prognosis was similar between sexes.
Jansen et al., 2021 [67]	266 ANOCA patients	85.7%	Epicardial CAS: >90% diameter reduction + chest pain + ischemic ECG changes. Microvascular CAS: <90% diameter reduction + chest pain + ischemic ECG changes	Men had more epicardial CAS and less microvascular CAS than women.
Park et al., 2022 [70]	5491 ANOCA patients	54.4%	Epicardial coronary spasm: >70% spasm + typical chest pain + ECG changes	5-year major clinical outcomes were similar between men and women.
Saito et al., 2022 [69]	797 ANOCA patients	46.4%	Epicardial CAS: angiographic spasm + chest pain + ischemic ECG changes	Men were more likely to have positive ACh provocation test as compared to women.
Rinaldi et al., 2025 [71]	519 patients with suspected CAS	53.0%	Epicardial CAS: >90% diameter reduction + typical chest pain + ischemic ECG changes. Microvascular CAS: <90% diameter reduction + typical chest pain + ischemic ECG changes	Women exhibited a higher incidence of microvascular CAS. In female patients, a positive ACh test was associated with a higher rate of angina recurrence.

Table legend: ACh: acetylcholine; ANOCA: angina with non-obstructive coronary artery disease; CAS: coronary artery spasm; CCBs: calcium channel blockers; LAD: left anterior descending artery; MACE: major adverse cardiovascular events; RCA: right coronary artery; VSA: vasospastic angina

## Abbreviations

The following abbreviations are used in this manuscript:

ACh	Acetylcholine
CAD	Coronary artery disease
CAS	Coronary artery spasm
CFR	Coronary flow reserve
CMD	Coronary microvascular dysfunction
COVADIS	Coronary Vasomotion Disorders International Study
IMR	Index of microvascular resistance
INOCA	Ischemia with non-obstructive coronary artery disease
MACE	Major adverse cardiovascular events
MVA	Microvascular angina
NTCVRF	Non-traditional cardiovascular risk factors
VSMC	Vascular smooth muscle cell
WISE	Women’s Ischemia Syndrome Evaluation
